# Helminth Glycans at the Host-Parasite Interface and Their Potential for Developing Novel Therapeutics

**DOI:** 10.3389/fmolb.2021.807821

**Published:** 2022-01-10

**Authors:** Myrna J. M. Bunte, Arjen Schots, Jan E. Kammenga, Ruud H. P. Wilbers

**Affiliations:** Laboratory of Nematology, Plant Sciences Group, Wageningen University and Research, Wageningen, Netherlands

**Keywords:** helminths, glycans, vaccines, immunomodulation, biopharmaceuticals

## Abstract

Helminths are parasitic worms that have successfully co-evolved with their host immune system to sustain long-term infections. Their successful parasitism is mainly facilitated by modulation of the host immune system *via* the release of excretory-secretory (ES) products covered with glycan motifs such as Lewis X, fucosylated LDN, phosphorylcholine and tyvelose. Evidence is accumulating that these glycans play key roles in different aspects of helminth infection including interactions with immune cells for recognition and evasion of host defences. Moreover, antigenic properties of glycans can be exploited for improving the efficacy of anti-helminthic vaccines. Here, we illustrate that glycans have the potential to open new avenues for the development of novel biopharmaceuticals and effective vaccines based on helminth glycoproteins.

## Introduction

Glycosylation is one of the most common post-translational modifications and contributes substantially to the molecular makeup of almost all living organisms. For infectious agents glycans play an important role in the interaction with their host in order to establish a successful infection. For example, glycosylation of the viral envelope proteins haemagglutinin and neuraminidase of influenza A impacts virus infectivity, virulence and host immune responses (Deshpande et al., 1987; Li et al., 1993; Schulze, 1997). Alternatively, impaired glycosylation of surface glycoproteins of the bacterium *Campylobacter jejuni* affects interactions with the host and results in reduced intestinal colonisation (Szymanski et al., 2002). For parasitic helminths, various glycans mediate the modulation of host immune responses, which benefits the establishment of long-term infections. Besides the advantages for the parasite, immunomodulatory effects could also be considered valuable for the host. Evidence is accumulating that areas where helminth infections are endemic are associated with a lower incidence of inflammatory diseases, like allergies and autoimmune diseases (Zaccone et al., 2006; Maizels and Yazdanbakhsh, 2008). The “hygiene hypothesis” and more recently the “old friends” hypothesis suggest that the absence of co-evolved microbes, including helminths, leads to defects in the establishment of immune regulatory networks (Rook, 2010). The lack of immunomodulatory effects in the absence of such infections could contribute to the development of inflammatory diseases, but also potentially immunological hyperactivity during SARS-CoV-2 (COVID-19) infection (Fonte et al., 2020; Hays et al., 2020; Gebrecherkos et al., 2021). Recent advances in glycobiology have contributed to further understanding of the interaction between glycosylated helminth products and the host immune system. Yet, there is still a lack of knowledge on the effects of many unique and complex helminth glycans on host immune responses. In this review we highlight the untapped potential of these unique helminth glycans for the development of successful helminth biopharmaceuticals and vaccines.

### Helminth Glycans and Their Role in PRR Signalling

Glycans on most helminth-released ES products play a role in the recognition by host innate immune cells. Upon infection, the glycan structures on ES products are recognised by pattern recognition receptors (PRRs), located on the surface of innate immune cells, such as dendritic cells (DCs) (Everts et al., 2010). For DCs, recognition and binding of an antigen to PRRs results in initiation of internal signalling pathways leading to the maturation of the DC itself. Mature DCs are able to release cytokines and mediate the polarisation of different T helper (Th) cell subsets including Th1, Th2, Th17 and regulatory T cells (Walsh and Mills, 2013).

**FIGURE 1:**
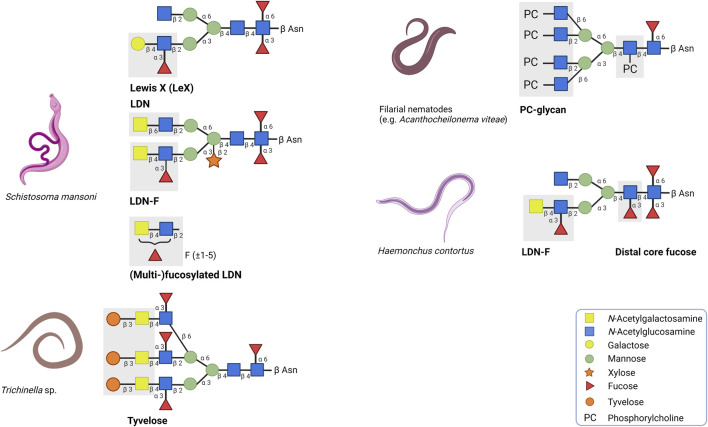
Overview of a selection of helminth glycans with strong immunogenic or immunomodulatory properties. The corresponding positions and symbols of the monosaccharide units are indicated. LDN and (multi)-fucosylated LDN found on secreted egg antigens of the trematode *Schistosoma mansoni* are associated with immunogenic responses whereas Lewis X has immunomodulatory effects. In contrast to the other glycan motifs, multi-fucosylated LDN is found on O-glycans. Tyvelose expressed by *Trichinella* species and distal core fucose expressed by *Haemonchus contortus* are glycan motifs which are associated to be important to induce humoral immunity. Glycoproteins carrying phosphorylcholine (PC) are mainly secreted by filarial nematodes and in many cases, PC is responsible for the immunomodulatory effects of these glycoproteins.

Different classes of PRRs, such as Toll-Like Receptors (TLRs) and C-type Lectin Receptors (CLRs), bind to specific glycan motifs and mediate internalisation to initiate an appropriate immune response. Antigen recognition by TLRs generally results in priming immature DCs to induce the production of pro-inflammatory cytokines and costimulatory molecules that contribute to the maturation of naive CD4^+^ T helper cells into Th1 or Th17 cells (Kapsenberg, 2003; Medzhitov, 2007; Weaver et al., 2007). However, TLR recognition of helminth antigens is associated with lower expression of pro-inflammatory cytokines and polarisation towards Th2 cells (MacDonald and Maizels, 2008). For example, the filarial nematode glycoprotein ES-62 interacts with TLR4 on immature DCs, thereby initiating MyD88-dependent signalling cascades that lead to maturation of DCs and cytokine release. Subsequently, mature DCs will promote the differentiation of Th2 cells (Goodridge et al., 2005). N-glycans of ES-62 are characterised by the presence of phosphorylcholine (PC) motifs (Figure 1) (Harnett et al., 2003). Studies using PC-conjugated ovalbumin (PC-OVA) demonstrated that PC-OVA mimics the effects of ES-62 on DCs in a TLR4- and MyD88-dependent manner (Goodridge et al., 2007). This suggests that the PC motif is responsible for the interaction of ES-62 with DCs via TLR ligation.

The CLR family of receptors have distinct binding properties to specific carbohydrate motifs (Weis et al., 1998; Zelensky and Gready, 2005). Carbohydrate structures are often bound in a calcium-dependent manner to the highly conserved carbohydrate recognition domain of the receptor. Dendritic cell-specific intracellular adhesion molecule-3-grabbing nonintegrin (DC-SIGN), macrophage galactose-type C-type lectin (MGL) and mannose receptor (MR) are the most prominent CLRs described regarding helminth glycoprotein recognition (Prasanphanich et al., 2013). DC-SIGN is highly expressed on the surface of DCs and can recognise the glycan motifs Lewis X (Galβ1-4(Fucα1-3)GlcNAc; LeX) and LDN-F (GalNAcβ1-4(Fucα1-3)GlcNAc), which are found on secreted egg antigens of the trematode *Schistosoma mansoni* (Figure 1) (van Die et al., 2003; van Liempt et al., 2007). LeX binding to DC-SIGN induces the production of the anti-inflammatory cytokine IL-10 and contributes to a modified Th2 response (Gringhuis et al., 2014; Wilbers et al., 2017). MGL binds and internalises glycoproteins with terminal β-GalNAc residues, such as LDN (GalNAcβ1-4GlcNAc) and LDN-F, whereas MR recognises mannose- and fucose-containing glycans (Taylor et al., 1992; Denda-Nagai et al., 2002; van Vliet et al., 2005). Binding of the LeX-carrying glycoprotein omega-1 by MR has demonstrated to facilitate binding and internalisation of omega-1 by DCs (Everts et al., 2012; Wilbers et al., 2017).

Although there are strong indications that glycans play an important role in immune recognition, for many helminth-derived glycoproteins it is still unknown how their glycans interact with immune cells and contribute to the modulation of the host immune system. Therefore, a deeper understanding is required of glycan interaction with PRRs and innate immune cells.

### Antibody Responses to Helminth Glycans

Many helminth-derived products can initiate a humoral response by inducing the generation of antibodies. A large part of these antibodies binds specifically to the glycan structures on ES products, indicating that glycans can serve as epitopes. In some cases, antibodies against these glycan epitopes reduce an infection. An example of such a glycan epitope is tyvelose (β3,6-dideoxy-D-arabinohexose), a glycan modification found on the glycoprotein TSL-1, which is excreted by *Trichinella spiralis* during the infective larval muscular phase (L1) (Figure 1) (Reason et al., 1994). During this phase, IgG antibody responses are induced that predominantly target the β-anomeric tyvelose on TSL-1 (Appleton et al., 1988; Appleton et al., 1991; Ellis et al., 1997). These anti-tyvelose antibodies may play an important role in host-protection. In an *in vitro* model with intestinal epithelial cells, it was demonstrated that the tyvelose-specific antibodies inhibit the invasion of *T. spiralis* (McVay et al., 1998; McVay et al., 2000). In addition, the presence of these antibodies causes a rapid expulsion of L1 larvae, eliminating 99% of a total oral challenge dose in rats (Appleton and McGregor, 1984; Appleton et al., 1988; Bell et al., 1992; Ellis et al., 1994). The induced immunity can provide long-term protection (up to several months) and can be transferred to offspring (Bullick et al., 1984; Appleton et al., 1985).

Although anti-tyvelose antibodies can induce high-level protection against L1 larvae, these antibodies are not providing protective immunity against intestinal stages of infection (Goyal et al., 2002). This led to the idea that the presence of tyvelose may also offer protection for the parasite, since the expression of tyvelose and its associated immune response is specific for L1-staged larvae (Denkers et al., 1990; Robinson et al., 1995). By the time the host immune system has generated the required antibodies, the parasite continued its development and lost the tyvelose epitope. In this way, the strong antibody response may serve as a smoke screen to divert the immune system away from effective immune responses. However, there is limited evidence supporting this theory, so more research is required to further study the role of tyvelose during infection and in establishing protective immunity. Apart from its role in protection, anti-tyvelose antibodies may offer possibilities in terms of diagnostics. Immobilised anti-tyvelose monoclonal antibodies can relatively easy capture the major secreted TSL-1 antigens with high specificity and high sensitivity, which makes the antibodies suitable to be incorporated into serodiagnosis of human *Trichinella* infection (Escalante et al., 2004).

In case of *Schistosoma mansoni* infection, the host immune system encounters various highly glycosylated helminth-derived antigens. Throughout the life cycle, S. mansoni expresses a wide variety of glycan motifs on its N- and O-glycans as well as glycolipids. These motifs include LDN, (multi-)fucosylated LDN and LeX (Smit et al., 2015). Especially (multi-)fucosylated LDN glycan motifs are highly immunogenic and are targeted by IgM antibodies (and to a lesser extent IgG antibodies) in S. mansoni infected people (van Remoortere et al., 2001; Kariuki et al., 2008; Prasanphanich et al., 2014)*.* Furthermore, primates vaccinated with radiation-attenuated (RA) cercariae exhibited high levels of IgM and IgG antibodies against multi-fucosylated glycan epitopes (Yang et al., 2019). However, compared to multi-fucosylated glycans, LDN-F and LeX induce lower levels of antibodies, primarily of the IgM class (van Remoortere et al., 2001; Naus et al., 2003; Van Remoortere et al., 2003). This reduced immunogenicity could be explained by the fact that expressed LDN-F and LeX motifs are shared with the host (Hokke and Deelder, 2001; Van Remoortere et al., 2003; Hokke and van Diepen, 2017). By mimicking host-like glycans, *S. mansoni* could have gained the opportunity to evade the host immune system to create a suitable micro-environment.

### The Role of Glycans in Helminth Immunomodulation

**FIGURE 2:**
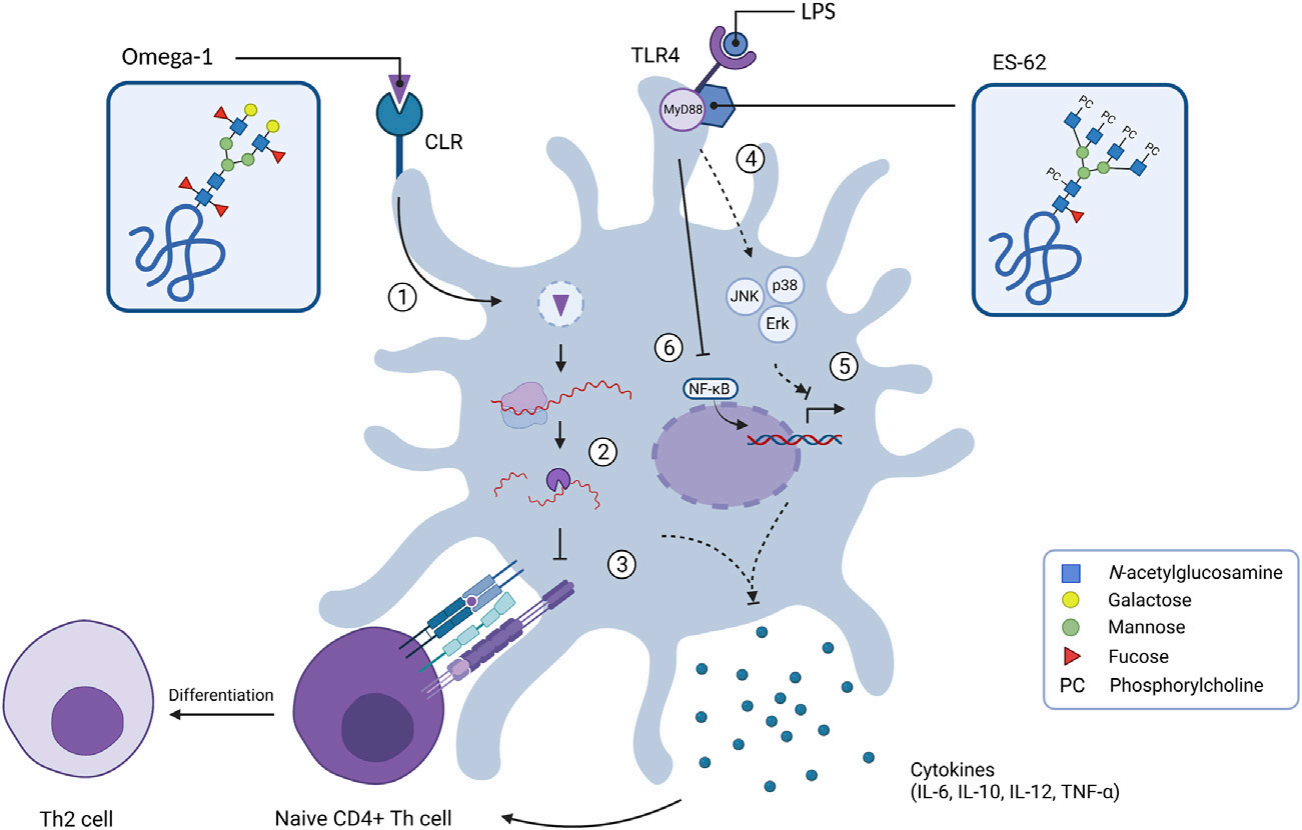
Internal signalling of dendritic cells upon exposure of glycoproteins omega-1 and ES-62. C-type lectin receptors can bind various glycan motifs such as LeX on omega-1, which results in uptake of the extracellular antigen. After internalisation in endosomes (1), omega-1 translocates to the cytosol where it interferes with protein translation by exhibiting RNase activity on messenger RNA and ribosomal RNA (2). This suppresses DC maturation by inhibiting IL-12 cytokine release whilst enhancing IL-10 production and down-regulation of co-stimulatory molecules such as CD86. This ultimately results in T helper 2 (Th2) cell polarisation (3). Glycoprotein ES-62 biases the immune system towards an anti-inflammatory/Th2 response as it is able to mediate the LPS-induced TLR4 signalling by regulating the activity of kinases Erk, JNK and p38 via adaptor molecule MyD88 (4). This leads to down-regulation of gene transcription of Th1-associated cytokines (IL-6, IL-12p38 subunit and TNF-α) (5). ES-62 inhibits the production of IL-12p40 subunit by preventing the binding of transcription factor NF-κB to the IL-12 promotor (6).

Immunomodulation serves as an important survival strategy for helminth parasites. Biasing the host immune system towards a modified type 2 immune response results in a more anti-inflammatory status, which decreases the chances of elimination. Glycans play an important role in the modulation of immune signalling *via* CLRs on DCs, thereby facilitating internalisation of helminth-released products and subsequent alteration of the immune response. For example, LeX mediates the internalisation of the *S. mansoni* egg antigen omega-1 into DCs *via* the MR and DC-SIGN receptors (Steinfelder et al., 2009; Everts et al., 2012; Wilbers et al., 2017). Within the cell omega-1 has RNase activity, which reduces the activation and maturation of DCs, thereby biasing towards a Th2 response (Figure 2). The immunomodulatory properties of the LeX motif by itself have also been investigated using lacto-*N*-fucopentaose III (LNFPIII), a LeX carrying penta-saccharide found in breast milk and urine of pregnant women (Ko et al., 1990). This LNFPIII glyco-conjugate promotes an anti-inflammatory response by the induction of alternatively activated macrophages (Atochina et al., 2008). Macrophage and DC activation by LNFPIII appears to be dependent on α1,3-linked fucose of the LeX motif, suggesting that the presence of α1,3-linked fucose is necessary for LNFPIII to exert its effects (Tundup et al., 2015).

In addition, human filarial nematodes, such as *Brugia malayi, Wuchereria bancrofi* and *Onchocerca volvulus* secrete phosphorylcholine (PC)-containing glycoproteins, which can drive immunomodulation (Forsyth et al., 1985; Harnett et al., 1989; Maizels et al., 1987). The best characterised PC-carrying glycoprotein is ES-62 and is found in the ES products of the rodent filarial nematode *Acanthocheilonema viteae* (Harnett et al., 1989)*.* ES-62 has immunomodulatory effects on a wide variety of immune cells including T and B cells, macrophages and DCs (Pineda et al., 2014). ES-62 polarizes the immune system towards a type 2 immune response by inducing Th2 cells instead of Th1 cells (Harnett et al., 1999). When the PC motif is removed, this Th2 polarisation is balanced out, suggesting that PC is predominantly responsible for the immunomodulatory effects of ES-62 (Houston et al., 2000). Interestingly, PC-conjugates mimic several of these immunomodulatory effects, such as the TLR4-dependent interaction with DCs (Goodridge et al., 2005). PC-conjugates also dampen the activation of DCs, which results in reduced expression of costimulatory molecules and Th1/Th17-polarising cytokines IL-12, IL-6 and TNF-α (Figure 2) (Goodridge et al., 2004; Goodridge et al., 2007; Lumb et al., 2017). In B cells, exposure to PC-conjugates results in interference of activation and proliferation of these immune cells (Harnett and Harnett, 1993). The presence of PC on ES-62 causes desensitisation of the B cell receptor (BCR) by inhibiting key proliferation pathways (Deehan et al., 2001). Mice exposed to PC-conjugates produced high levels of anti-inflammatory cytokine IL-10 and IgM antibodies, which are also abundant during filarial nematode infections (Al-Qaoud et al., 1998). Therefore, it is reasonable to state that PC by itself is acting as an active ingredient on ES-62 as it is largely mediating the immunomodulatory effects of this helminth ES product.

Overall, these examples indicate that glycan motifs such as LeX and PC have broad immunomodulatory properties, which may be interesting for the development of biopharmaceuticals. Attempts have been made to explore the potential of helminth-derived glycoproteins to serve as therapeutic agents. For example, plant-produced recombinant omega-1 with LeX-carrying glycans was able to reduce body weight, fat mass and food intake of obese mice and improve their insulin sensitivity and glucose tolerance (van der Zande et al., 2021). In addition, immunomodulatory effects of the PC-glycoprotein ES-62 led to the design and synthesis of small molecule analogues (SMAs) that mimicked the chemical structure of PC (Al-Riyami et al., 2013). Besides providing additional evidence that immunomodulatory properties of ES-62 depend on PC, these SMAs offer solutions for treatment against immune disorders. Studies in mouse models of inflammation-associated diseases such as rheumatoid arthritis, systemic lupus erythematosus and asthma demonstrated that these SMAs possess anti-inflammatory properties similar to ES-62 (Al-Riyami et al., 2013; Rzepecka et al., 2014; Al-Riyami et al., 2015; Rzepecka et al., 2015; Coltherd et al., 2016; Janicova et al., 2016; Lumb et al., 2017; Doonan et al., 2018; Suckling et al., 2018). Since ES-62 is immunogenic and hard to obtain on large scale, SMAs exhibit great therapeutic potential against inflammation-associated diseases.

### Challenges and Opportunities of Glycans in Helminth Vaccine Development

To date, helminths still pose a serious threat for human and animal health. The current treatment of human and animal helminth infections primarily consists of anthelminthic drugs in mass drug administration (MDA) programs. However, anthelminthic drugs are never fully effective to eradicate helminths from their host, so re-administration is necessary. This may result in reduced efficacy through the development of resistance against anthelminthic drugs (De Clercq et al., 1997; Ismail et al., 1999; Melman et al., 2009; Osei-Atweneboana et al., 2011; Sutherland and Leathwick, 2011; Nana-Djeunga et al., 2014; Crellen et al., 2016). For this reason, vaccines against helminth infections are proposed as a more effective and durable treatment.

Given that some glycans seem to induce a potent immune response, the idea has arisen that helminth-derived glycans aid in the efficacy vaccines. Attempts have been made to develop a vaccine against *Schistosoma mansoni* by using radiation-attenuated (RA) cercariae. Vaccination of primates with RA cercariae demonstrated to provide protection against *Schistosoma* infection. In vaccinated chimpanzees, the faecal egg count and the level of circulating cathodic antigens (CCA) were reduced by 38 and 46%, respectively, compared to non-vaccinated control animals (Eberl et al., 2001a). Baboons vaccinated with RA cercariae developed even higher protection since their worm burden, faecal egg count and CCA were reduced by up to 86, 94 and 99%, respectively, compared to non-vaccinated baboons (Kariuki et al., 2004). This induced protection correlates with high levels of specific IgM and IgG antibodies targeting RA cercariae-derived glycan epitopes (Eberl et al., 2001b; Yang et al., 2019). High titres of IgG against (multi-) fucosylated glycan epitopes were observed in RA vaccinated baboons (Yang et al., 2019). These IgG levels remained high until 6 weeks post-challenge, suggesting a potent role of these fucose-targeting IgG antibodies in protection. Although, these results are promising, the induced protection could not be sustained for long-term (Kariuki et al., 2004). Therefore, more research is required to improve sustained efficacy of RA cercariae as vaccine.

To combat livestock-infecting helminths, only three commercial vaccines are available on the market but many vaccines are still in development (Claerebout and Geldhof, 2020). Many helminth vaccines rely on life-attenuated parasites, parasite extracts or purified native antigens, which require the use of donor animals to produce the important ingredients of the vaccines. Besides ethical issues it is challenging to collect enough parasite material for large-scale vaccine production. Therefore, the use of recombinant production systems has been explored, resulting in more than 100 vaccine studies with recombinant helminth antigens, including 80 different antigens targeting 22 helminth species (Geldhof et al., 2007). However, only few recombinant vaccines induce high-level protection against helminth infection (Geldhof et al., 2008; Hewitson and Maizels, 2014). The choice for the bacterium *Escherichia coli* as recombinant expression system could be a possible explanation why most recombinant helminth vaccines fail to induce high-level protection, since *E. coli* is incapable to perform complex eukaryotic post-translational modifications like glycosylation. Therefore, other eukaryotic expression systems have been considered to produce recombinant helminth vaccines with complex post-translational modifications.

For example, different expression systems have been investigated for the production of recombinant activation-associated secreted protein 1 of *Ostertagia ostertagi* (OoASP-1). Vaccination of cows with a native ASP-enriched fraction made from an ES-thiol fraction, of which OoASP-1 and OoASP-2 are the most abundant antigens, resulted in 74% reduction of faecal egg counts (Geldhof et al., 2002; Geldhof et al., 2003; Meyvis et al., 2007). To convert this native vaccine to a recombinant version, expression of OoASP-1 in the yeast *Pichia pastoris* and the baculovirus insect cell system was explored. *Pichia pastoris*-expressed OoASP-1 showed a less potent immune response than native OoASP-1 and both recombinant OoASP-1 variants failed to induce protection upon re-infection (Geldhof et al., 2008; González-Hernández et al., 2016). A reason for this could be that native OoASP-1 is differently glycosylated in recombinant systems. Incorrect glycosylation could impact potentially important glycan epitopes, the correct folding of the protein or the interaction with immune cells. Removal of the glycans of the ES-thiol fraction demonstrated that antibody binding to these antigens is not affected by the lack of glycosylation, whereas changing protein confirmation by denaturation/reduction did (Meyvis et al., 2008). Improper folding of recombinant OoASP-1 by an altered glycan composition could therefore result in unreachable epitopes, which might be required for inducing protective immune responses.

The development of recombinant vaccines against *Haemonchus contortus* infection has faced similar challenges. Currently, the *H. contortus* vaccine Barbervax is approved and marketed in Australia and is based on ES material enriched with glycoproteins H11 and H-gal-GP (Nisbet et al., 2016). The production of Barbarvax relies on conventional isolation of ES material from parasites from infected donor sheep. Therefore, other strategies to employ *H. contortus* glycoproteins as recombinant vaccine candidates have been explored. Glycoprotein H11 has been subjected to vaccine trials as vaccination with an extract enriched with H11 demonstrated high levels of protection and the generated antibodies were mostly directed towards H11 (Munn et al., 1993). Initial vaccine trials using recombinant H11 produced in *E. coli* and baculovirus insect cell system were unsuccessful to induce protection (Knox et al., 2003; Newton and Meeusen, 2003; Reszka et al., 2007). Failure has mainly been directed to the differences in post-translational modifications that impacted the glycosylation and folding of H11. Mass spectrometry analysis showed that N-glycans of H11 are highly fucosylated and contain α1,3- and α1,6-linked core fucoses on the proximal GlcNAc and α1,3-linked fucose on the distal core GlcNAc (Figure 1) (Haslam et al., 1996). Most of the generated antibodies were directed against these H11 glycan epitopes. Moreover, α1,3-linked core fucose induces the production of IgE antibodies, which correlated with the induction of protection (van Die et al., 1999; Kooyman et al., 2000). This suggests that α1,3-linked core fucose serves as an important epitope to induce humoral immunity. To test whether recombinant fucosylated H11 induces protection, H11 was expressed in the free-living nematode *Caenorhabditis elegans* since it has the required glycan machinery to mimic the native glycan composition of H11 (Nguyen et al., 2007; Roberts et al., 2013). Although *C. elegans* expressed H11 carried N-glycans with α1,3- and α1,6-linked core fucoses and α1,3-linked distal core fucose, no protection was observed against *H. contortus* challenge infection (Roberts et al., 2013). The lack of efficacy could be attributed to subtle differences in the glycan composition of H11 upon expression in *C. elegans*. For instance, *C. elegans* expressed H11 lacks LDN-F, a glycan motif previously shown to be correlated with protective immunity against *H. contortus* in lambs (Vervelde et al., 2003). Similarly, antibody responses to the O-glycan motif Galα1–3GalNAc glycan epitopes correlates with protection in lambs that are vaccinated with ES products of *H. contortus* (van Stijn et al., 2010)*.*

Differences in glycan composition or glycan abundance could be the reason why recombinant vaccines against helminths are not able to induce protective immunity. Therefore, further mimicking the native glycan composition could improve the immunogenicity towards inducing sufficient protection.

## Conclusion

There is substantial evidence that helminth glycans play a key role in establishing immunomodulatory and protective immune responses by the host. Although recent efforts have gained new insights on the role of several helminth glycans at the host-parasite interface, many questions remain to be addressed. Technological advances in the field of glycobiology, including mass spectrometry-based glycomics, glycan-arrays and glyco-engineering, will allow us to unravel the complexity of helminth glycomes. In parallel, there is a need for recombinant expression systems that are able to produce helminth glycoproteins with a native glycan composition in order to study their role in immunogenicity and/or immunomodulation. Plants could serve as such expression system as it offers “engineering” of the post-translational machinery, resulting in tailor-made helminth glycan structures (Wilbers et al., 2017; Van Noort et al., 2020). Altogether this offers opportunities to exploit the untapped potential of helminth glycans to develop helminth-derived biopharmaceuticals, diagnostics and improved vaccines.
